# MRI radiomics predicts progression-free survival in prostate cancer

**DOI:** 10.3389/fonc.2022.974257

**Published:** 2022-08-30

**Authors:** Yushan Jia, Shuai Quan, Jialiang Ren, Hui Wu, Aishi Liu, Yang Gao, Fene Hao, Zhenxing Yang, Tong Zhang, He Hu

**Affiliations:** ^1^ Affiliated Hospital, Inner Mongolia Medical University, Hohhot, China; ^2^ Department of Pharmaceuticals Diagnosis, GE Healthcare (China), Shanghai, China; ^3^ Department of Radiology, Affiliated Hospital of Inner Mongolia Medical University, Hohhot, China

**Keywords:** prostate cancer, radiomics, progression-free survival, magnetic resonance imaging, predictions

## Abstract

**Objective:**

To assess the predictive value of magnetic resonance imaging (MRI) radiomics for progression-free survival (PFS) in patients with prostate cancer (PCa).

**Methods:**

191 patients with prostate cancer confirmed by puncture biopsy or surgical pathology were included in this retrospective study, including 133 in the training group and 58 in the validation group. All patients underwent T2WI and DWI serial scans. Three radiomics models were constructed using univariate logistic regression and Gradient Boosting Decision Tree(GBDT) for feature screening, followed by Cox risk regression to construct a mixed model combining radiomics features and clinicopathological risk factors and to draw a nomogram. The performance of the models was evaluated by receiver operating characteristic curve (ROC), calibration curve and decision curve analysis. The Kaplan-Meier method was applied for survival analysis.

**Results:**

Compared with the radiomics model, the hybrid model consisting of a combination of radiomics features and clinical data performed the best in predicting PFS in PCa patients, with AUCs of 0.926 and 0.917 in the training and validation groups, respectively. Decision curve analysis showed that the radiomics nomogram had good clinical application and the calibration curve proved to have good stability. Survival curves showed that PFS was shorter in the high-risk group than in the low-risk group.

**Conclusion:**

The hybrid model constructed from radiomics and clinical data showed excellent performance in predicting PFS in prostate cancer patients. The nomogram provides a non-invasive diagnostic tool for risk stratification of clinical patients.

## Introduction

Prostate cancer is the most common malignancy of the male reproductive system, the fourth most common cancer worldwide, and the fifth leading cause of cancer death in men (1, 2). There are significant geographical differences in its incidence. With economic development and increased life expectancy, the incidence and mortality of PCa are on the rise in Asian countries, with an increasing disease burden (3). According to the US Surveillance, Epidemiology and End Results (SEER) Database 2010-2016 data, the 5-year survival rate for metastatic PCa is only 30% (4). The onset of PCa is insidious, and most patients are already at intermediate to the advanced risk of PCa at the time of initial diagnosis, with a high rate of recurrence and risk of metastasis (5). Therefore, it is particularly important to find a suitable way to predict the progression of prostate cancer patients and intervene early to prolong their survival.

Artificial intelligence (AI), the ability of machines to perform cognitive tasks to achieve specific goals based on the data provided, is transforming our healthcare system. Machine learning (ML) is a subfield of AI, meaning that algorithms are created and deployed to analyze data and its properties, and are not specifically given tasks based on certain predefined inputs in the environment. In order to improve the probability of survival of prostate cancer patients, it is necessary to develop appropriate predictive models for PCa. Jović S et al. (6) applied and compared several machine learning techniques in their study for analytical discussion and concluded that machine learning techniques can be used for prediction related to prostate cancer. The use of computer-based learning models has become a major area of research in PCa. Conventional imaging is usually used for diagnosis, staging and treatment guidance of tumors and the information obtained from the images is subjective. Dutch scholar Lambin (7) first introduced the concept of radiomics in 2012, which promises to visualize heterogeneity within tumors and reveal the prognostic information behind the images. It builds on imaging techniques such as magnetic resonance imaging (MRI), computed tomography and positron emission tomography to convert medical images into high-dimensional, mineable data through high-throughput extraction of quantitative features, thereby providing decision support for oncology at low cost and non-invasively (8). Ferro M et al. (9) summarize the latest studies using different imaging modalities, following a predefined methodology, looking for studies with validated protocols, but also looking at how AI can improve radiomics and translate these results into clinical practice, and about the advantages and limitations of the different algorithms used in PCa radiomics. In addition, many studies in recent years have shown that radiomic features are related to molecular features of cancer tissue, genomics, proteomics and metabolomics (10). This new area of research in PCa is an extension of radiomics, whose main focus is on tailored approaches to diagnose aggressive PCa (11), predict prognosis (12), progression (13) and response to treatment (11). MRI with its high soft tissue resolution and multidirectional imaging capabilities can clearly show the different locations of lesions in prostate cancer, and in combination with functional imaging plays an important role in assessing the presence of extra capsular extension (ECE), seminal vesicle invasion, in prostate cancer detection (14), staging (15) and aggressiveness assessment (16) and is the most commonly used imaging modality in prostate cancer screening. A number of published findings support mp-MRI (17, 18) as the most sensitive and specific imaging modality.

Progression-free survival is important for the prognostic assessment of tumor patients, and studies have demonstrated that radiomics can be used to predict progression-free survival in glioma (19), breast cancer (20), lung cancer (21) and ovarian cancer (22), but to date, no personalized imaging prediction models have been developed for progression-free survival in prostate cancer patients. Therefore, this study evaluates the value of MRI radiomics in predicting progression-free survival in PCa patients to develop a hybrid clinical-imaging histology model to help improve decision-making and guide individualized treatment.

## Material and methods

### Patient selection

**Figure 1:**
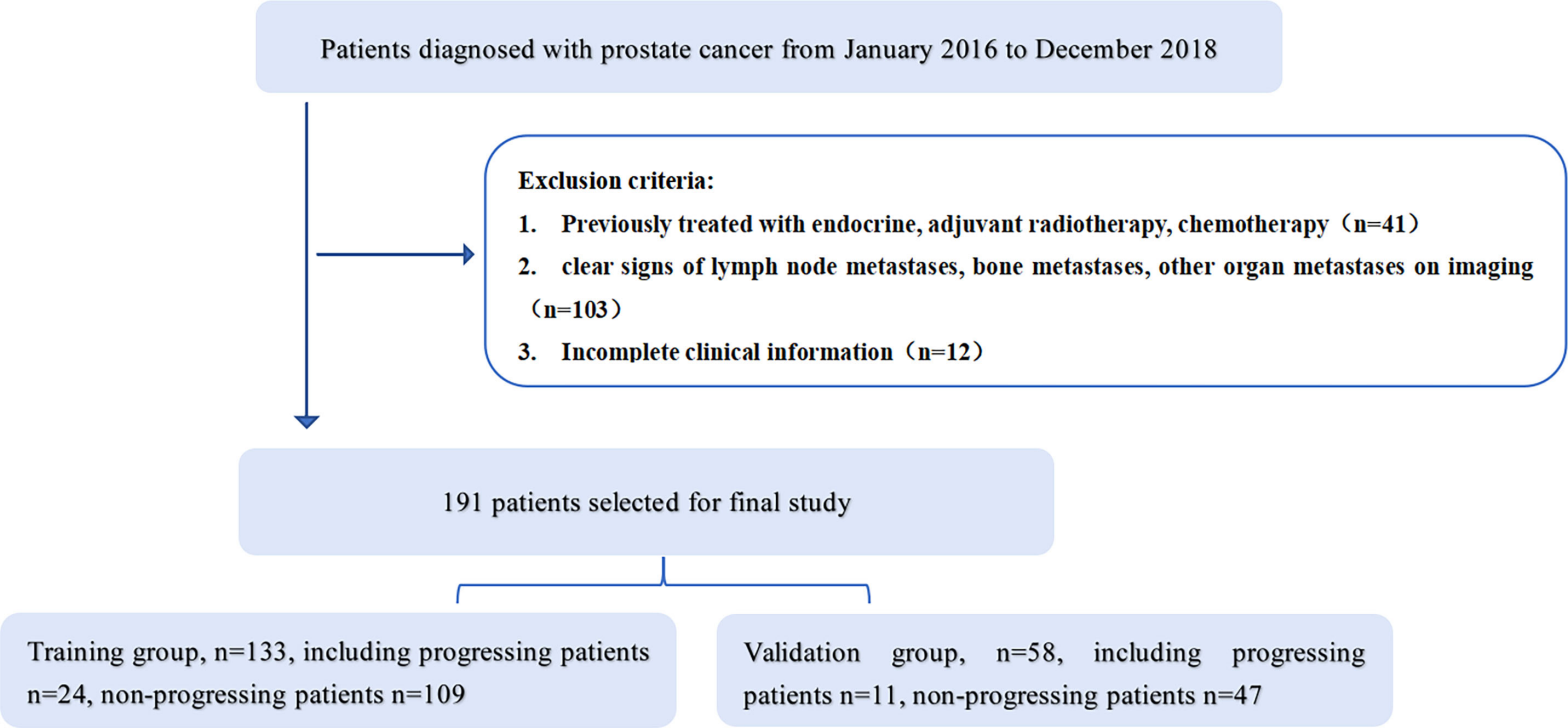
Patient selection flow chart. Includes exclusion criteria and grouping.

This study was approved by the Ethics Committee of the Affiliated Hospital of Inner Mongolia Medical University, and informed consent was obtained from patients. A retrospective collection of 373 patients with PCa retrieved from our hospital’s image archiving and communication system (PACS, GE) from January 2016 to December 2018 was conducted. Patient groupings are shown in **Figure 1**. Inclusion criteria: 1. Patients with histologically confirmed T1-4N0M0 prostate cancer confirmed by puncture biopsy or surgical pathology; 2. Undergoing MRI one week prior to treatment. Exclusion criteria: 1. previous endocrine, radiotherapy or chemotherapy; 2. clear signs of metastasis on MRI; 3. incomplete clinical profile. The final 191 patients were included in the study, (aged 45-89 years, median age 74 years) and were randomised in a 7:3 ratio into a training group (n=133) and a validation group (n=58). Clinical information on all patients included age, pre-treatment PSA levels, number of lesions, clinical T-stage and Gleason score.

All patients are followed up at 3 months for 2 years, every 6 months after 2 years and once a year after 5 years. The follow-up deadline is December 2021. Follow-up visits include PSA levels, CT of the chest, abdomen and pelvis or MRI of the pelvis, and bone scans. The endpoint is progression-free survival, defined as the time from the first day of treatment until disease progression (biochemical recurrence, distant metastases, including bone metastases, lymph node metastases and other distant organ metastases) or death from any cause, or the last follow-up visit.

### MRI acquisition

All scans were performed using a GE Discovery MR 750 3.0T superconducting MRI machine with an abdominal coil in all patients. The acquisition parameters were as follows: axial T2- weighted spin-echo images (repetition time/echo time [TR/TE]: 3,480/85 ms, field of view[FOV] = 24 cm, matrixs = 320x320, lice thickness = 4 mm, spacing = 1.0 mm), axial T1-weighted spin-echo images (TR/TE: 811/10 ms, FOV = 24 cm, matrixs = 320x224, slice thickness = 4 mm, spacing = 1.0 mm), and axial DWI SE-EPI images (TR/TE: 2,900/61, FOV = 28 cm, matrixs = 512x512, slice thickness = 4 mm, spacing = 1.0 mm, b = 0, 1,000 s/mm^2)^. ADC maps were obtained in GE AW 4.6 Functool workstation post-processing.

### Image segmentation

**Figure 2:**
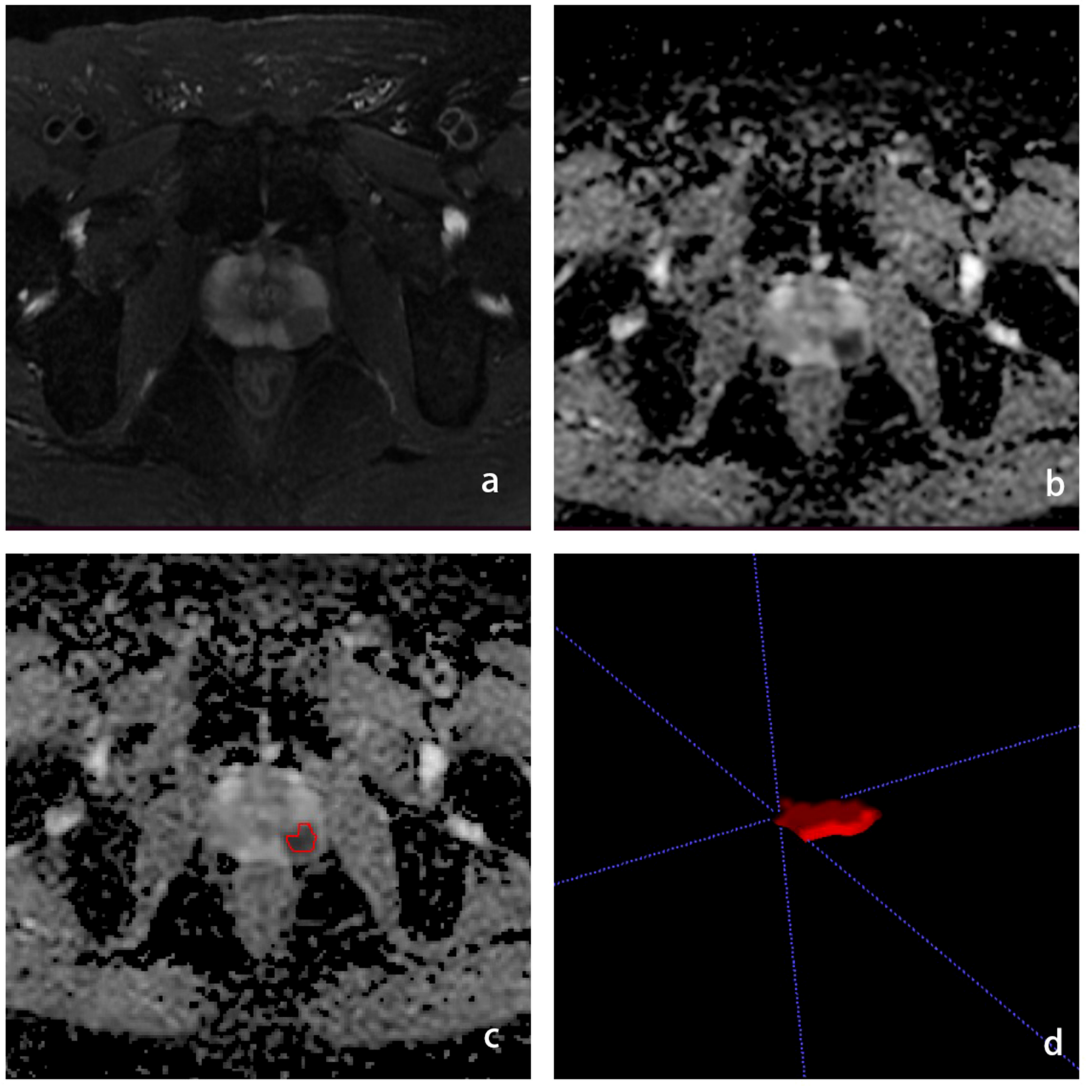
Schematic diagram of the ROI outline. **(A)** is the T2WI sequence with PCa in the left peripheral band, **(B)** is the ADC sequence with the cancer foci showing low signal, **(C)** is the ROI outline, **(D)** is the generated ROI.

We used the open-source software ITK-SNAP software for lesion segmentation. Radiologists with 5 years of experience in male pelvic MRI imaging were used to outline ROIs along the edges of the lesion at the largest level of the lesion on T2WI and ADC images, respectively, avoiding fat, calcifications and hemorrhagic foci. To select robust features for intra-rater and inter-rater description variation, intra-rater test datasets and intra-rater test datasets were obtained for 50 patients (**blind** with 15 years of experience in urological imaging) by the same radiologist and another radiologist, respectively (**Figure 2**).

### Extraction and selection of radiomics features

From each Roi, radiomic features were extracted from DWI, ADC images using the open-source tool pyradiomics. These features include: 1. Shape features: used to describe the geometric properties of the ROI, including size elements that describe the volume and surface area of the ROI. 2. First-order features, which are features describing the intensity distribution of voxels within the ROI, calculated by histogram analysis. 3.Texture features that describe the intensity level of the spatial distribution of voxels. Includes Grey Level Co-occurrence Matrix (GLCM) features, Grey Level Travel Length Matrix (GLRLM) features and Grey Level Size Zone Matrix (GLSZM) features.4. Algorithmically transformed features: first-order and higher-order texture features obtained by transforming the original image with Wavelet and Laplacian-of-Gaussian (LOG). 1307 radiomic features were extracted from each ROI.

### Construction of radiomics signatures

First, features with low repeatability were excluded from the subsequent analysis. Here the intra-rater and inter-rater repeatability for each feature was quantified by intraclass correlation coefficient (ICC) calculated on the intra-rater test data set and inter-rater test data set respectively. Features with ICC > 0.8 are retained. All features were normalized using the Z-Score transform. Single-factor logistic regression and GBDT were then used to further filter the histological features to ensure reproducibility of the model and reduce overfitting or selection bias in the radiomics model. The screened radiomics features were analyzed using Cox risk regression to create a radiomics model. Significant clinical variables were screened using univariate Cox risk regression. ROC curves, calibration curves, and decision curve analysis were applied to assess model performance.

### Validation of radiomics signatures

Kaplan-Meier survival analysis was used in the training group to assess the potential association of radiomic features with PFS, which was then validated in the validation group. Classification of patients into high and low risk groups based on cut-off values based on radiomic signatures as determined by optimal cut-off analysis using X-title software. The truncation values are estimated on the training group and validated on the validation group. A weighted log-rank test was used to assess the difference in survival curves between the high and low risk groups. To demonstrate the value of radiomic features for individualized assessment of PFS, separate radiomic column line plots were constructed. Radiomics scores (Rad scores) and clinical data were combined to create a mixed model of radiomics and clinical data to plot nomograms and provide a visual tool for predicting progression-free survival in PCa. The Rad score is calculated by adding selected imaging histology features that are weighted by their respective coefficients. Significant clinical variables were screened using univariate Cox risk regression.

### Statistical analysis

All statistical analyses for this study were performed using R software (Version 3.6.3, Statistical Computing Basis). A two-sided P < 0.05 was considered statistically significant. The Kolmogorov-Smimov test was used to verify that the histological characteristics conformed to a normal distribution, using the two independent samples t-test for normal distribution and the Mann-Whitney U test for non-normal distribution. The ability of the model was assessed by the ROC, calculating the AUC and 95% confidence intervals. The diagnostic sensitivity, specificity, accuracy, positive predictive value and negative predictive value of the models were also calculated. Calibration curves were used to assess the predictive performance of each model. Decision curves were used to assess the net benefit of each model at different threshold probabilities and to evaluate the clinical applicability of each model.

## Results

### Clinical data

Clinical data for patients in the training and validation groups are shown in **Table 1**. Patients were aged 45-89 years, with a median age of 74 years. The median progression-free survival time was 42 months (range 10-72 months). There was no statistically significant difference between the training and validation groups in terms of patient age (p > 0.05) and statistically significant differences in Gleason score, clinical T-stage, number of lesions and pre-treatment PSA levels (p < 0.05).

**Table 1:** Comparison of clinical characteristics between the training and validation groups.

Clinical data	Training group	Validation group	*P*
	n=133	n=58	
Age (mean ± SD, years)	72.12 ± 8.82	73.31 ± 8.40	0.765
T stage			0.001
T1	47	15	
T2	53	32	
T3	15	6	
T4	18	5	
Pre-treatment PSA levels(n/ml)			0.001
<100	67	30	
>100	66	28	
Gleason Score			0.001
5	12	2	
6	23	11	
7	43	22	
8	29	13	
9	16	6	
10	10	4	
Number of tumors			0.013
=1	86	31	
>1	47	27	

SD, standard deviation; PSA, prostate specific antigen.

### Radiomic signature building

**Figure 3:**
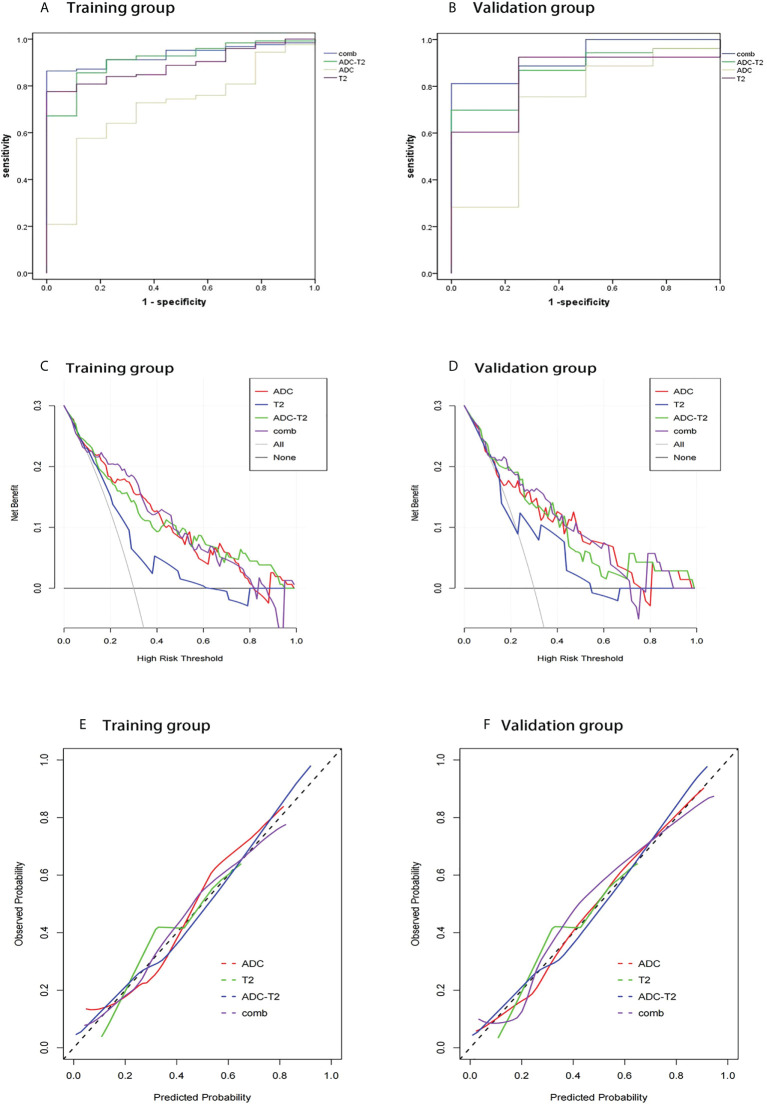
ROC curves, decision curve analysis, calibration curves for different models in the training and validation groups. The ROC curves for the four models in the training and validation groups are shown in **(A, B)**. The decision curves for the four models in the training and validation groups are shown in **(C, D)**. The calibration curves for the four models are shown in **(E, F)**.

1037 radiomic features were extracted from the ROI, and after t-test or Mann-WhitneyU test screening to remove the meaningless features, 5 optimal features were finally obtained from T2W1 and 4 optimal features from ADC using single factor logistic regression and the GBDT method, and the feature screening results are shown in **Table 2**. The results show that the hybrid model has better predictive ability, and the ROC curves of the four models in the training and validation groups are shown in **Figures 3A, B**. The AUCs of the T2WI, ADC, T2WI-ADC models and the hybrid model in the training group are 0.876 (0.815, 0.931), 0.722 (0.562, 0.856), 0.904 (0.833, 0.965), 0.904 (0.833, 0.965), and 0.926 (0.882, 0.962), and the AUCs in the validation group were 0.843 (0.673, 0.965), 0.713 (0.444, 0.945), 0.870 (0.75, 0.972), and 0.917 (0.808, 1.0), respectively (shown in **Table 3**). The four model decision curves and calibration curves are shown in **Figures 3C–F**.

**Table 2:** Radiomic feature selection result.

Classification of results	T2WI	ADC	T2WI-ADC
Number of features	5	4	9
Radiomics Features	log-sigma-5-0-mm-3D_firstorder_10Percentile	wavelet-LLL_firstorder_InterquartileRange	log-sigma-5-0-mm-3D_firstorder_10Percentile
	Wavelet-LHL_gldm_SmallDependenceHighGrayLevelEmphasis	wavelet-LLL_glszm_SmallAreaHighGrayLevelEmphasis	wavelet-LHL_gldm_SmallDependenceHighGrayLevelEmphasis
	wavelet-HLL_glcm_Correlation	original_glrlm_GrayLevelNonUniformityNormalized	wavelet-HLL_glcm_Correlation
	Wavelet-LHL_glcm_MaximumProbability	wavelet-LHH_glrlm_RunEntropy	wavelet-LHL_glcm_MaximumProbability
	log-sigma-3-0-mm-3D_firstorder_Minimum		log-sigma-3-0-mm-3D_firstorder_Minimum
			wavelet-LLL_firstorder_InterquartileRange
			wavelet-LLL_glszm_SmallAreaHighGrayLevelEmphasis
			original_glrlm_GrayLevelNonUniformityNormalized
			wavelet-LHH_glrlm_RunEntropy

T2WI, T2- weightedimagine; ADC, apparent diffusion coeffificient.

**Table 3:** Predictive performance of T2WI, ADC, T2WI-ADC and hybrid models.

Cohort	Model	AUC(95%CI)	ACC	SEN	SPE	PPV	NPV
Training	ADC	0.722(0.562,0.850)	0.729	0.728	0.750	0.978	0.150
	T2WI	0.876(0.815,0.930)	0.782	0.768	1.000	1.000	0.216
	T2WI-ADC	0.904(0.833,0.960)	0.850	0.848	0.875	0.991	0.269
	Hybrid models	0.926(0.882,0.960)	0.865	0.856	1.000	1.000	0.308
Validation组	ADC	0.713(0.444,0.940)	0.741	0.741	0.750	0.976	0.176
	T2WI	0.843(0.673,0.960)	0.707	0.704	0.750	0.974	0.158
	T2WI-ADC	0.870(0.750,0.972)	0.810	0.815	0.750	0.978	0.231
	Hybrid models	0.917(0.808, 1.000)	0.793	0.778	1.000	1.000	0.250

T2WI, T2- weightedimagine; ADC, apparent diffusion coeffificient; AUC, area under curve; SEN, sensitivity; SPE, specificity; ACC, accuracy; PPV, positive predictive value; NPV, negative predictive.

### Radiomics scoring and normogram creation

**Figure 4:**
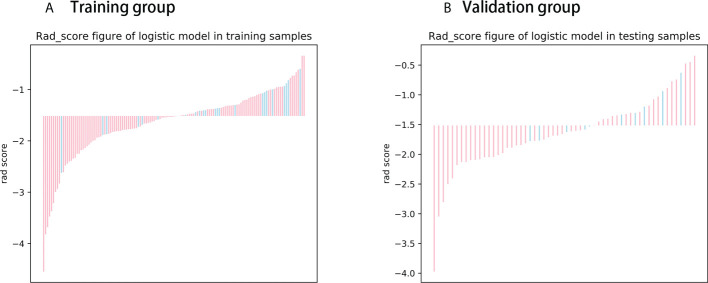
Rad score chart for training and validation groups. **(A, B)** show the distribution of radiomics scores for the training and validation groups respectively. The pink bars represent the radiomics scores of patients who did not experience disease progression, while the blue bars represent the radiomics scores of patients who experienced disease progression.

**Figure 5:**
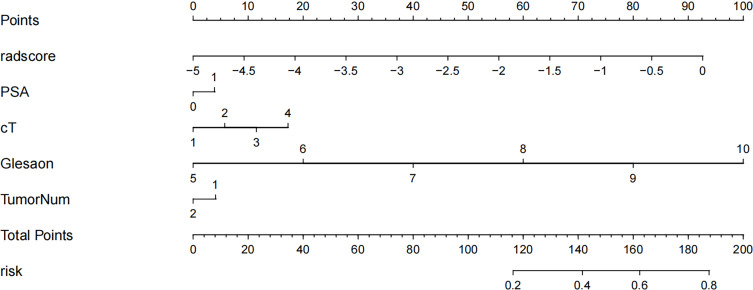
Radiology nomogram. The radiology nomogram prediction model predicts the probability of progression in patients with PCa. How to use: (1) locate the patient’s radiomic score, PSA level, clinical T-stage, Gleason score, number of tumor and then draw a straight line on the top dot axis to obtain the corresponding score; (2) sum the scores obtained (3) find the final sum on the total point axis and draw a straight line down to assess the risk of progression in patients with prostate cancer.

The Rad score was obtained by weighting the nine optimal features by their respective coefficients, calculated as = -1.6371 + 0.3323 × “ log-sigma-5-0-mm-3D_firstorder_10Percentile “-0.1502 ×“wavelet-LHL_gldm_SmallDependenceHighGrayLevelEmphasis “+ 0.1918 ×“ wavelet-HLL_glcm_Correlation”+ 0.3284” wavelet-LHL_glcm_MaximumProbability”+ 0.5209 × “log-sigma-3-0-mm-3D_firstorder_Minimum “- 0.5178 × “ wavelet-LLL_firstorder_InterquartileRange “+ 0.0487 ×“ wavelet-LLL_glszm_SmallAreaHighGrayLevelEmphasis “- 0.4251 ×“ original_glrlm_GrayLevelNonUniformityNormalized “- 0.3291 ×“ wavelet-LHH_glrlm_RunEntropy”. The Rad score plots for the training and validation groups are shown in **Figure 4**. Independent clinical predictors combined with Rad scores make up the Nomogram, as shown in **Figure 5**.

### Survival analysis

**Figure 6:**
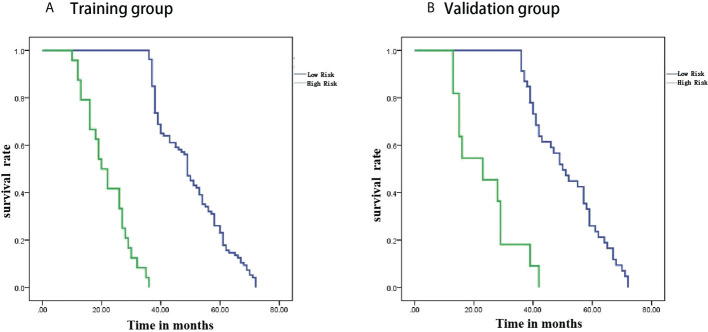
Kaplan-Meier analysis. **(A)** is the training group and **(B)** is the validation group.

Patients were divided into high-risk and low-risk groups based on radiomics scores. PFS survival curves were plotted using the Kaplan-Meier method. Using the log-rank chi-square test, there was a statistically significant difference in survival rates between the different risk groups in the training and validation groups (p<0.001) (**Figure 6**).

## Discussion

PCa is a common malignancy in elderly men, and its incidence and mortality are on the rise in some countries, especially in Asia. The insidious onset of PCa and the fact that it is mostly mid-to late-stage when first diagnosed has led to a decline in patient survival. Prognostic models associated with PFS have been developed in other tumor types with promising applications; however, according to our literature search, prognostic survival models for PFS imaging of PCa have not been studied. Imaging is an important clinical examination tool for diagnosis, staging and treatment decisions for tumors but relies heavily on the physician’s visual assessment of the images, which is subjectively biased and produces limited information. With the increased digitization of clinical information and the application of artificial intelligence research, radiomics has become a hot research topic. Solid tumors are spatially and temporally heterogeneous, and imaging histology can capture this heterogeneity noninvasively and express it in terms of pixel density and spatial distribution, which may correlate with tumor aggressiveness, pathological grading, posttreatment response and prognosis (7, 23, 24). In contrast, PCa is characterized by its remarkable heterogeneity and the variability of tumor prognosis. Most prostate cancers are inert, while the remaining proportion can be very aggressive and even life-threatening, so stratified management of patients with prostate cancer, early detection and effective intervention in high-risk patients to reduce recurrence and metastasis are important goals of current clinical research. Reliable and accurate predictors and prognostic models can help guide clinical decision-making to the clinical benefit of patients. In this context, we extracted features from MRI, constructed models and combined them with clinical factors to create nomograms for the further risk assessment of prostate cancer patients.

MRI-based radiomics have been extensively used in the diagnosis of prostate cancer, the Gleason score and other areas with satisfactory results (25–28). Recently, MRI radiomics has also been used to predict the risk of biochemical recurrence (BCR) after radical prostate cancer surgery and radiotherapy. BCR is considered a marker of local recurrence, distant metastasis and prostate cancer-specific death. Studies have reported (29) that the 10-year BCR rate after radical prostatectomy is as high as 50%. Gnep et al. (30) previously demonstrated in that Haralick features from T2WI were associated with BCR occurrence, suggesting that radiomics analysis may be able to capture the difference between BCR-positive and BCR-negative lesions on MRI. However, the role of MRI-based radiomics in assessing PFS in PCa has not yet been reported, so we have undertaken a study to investigate this. We used T2WI and ADC sequences to extract features because T2WI can clearly show the anatomical features of the tumor and the presence of perineural involvement and seminal gland involvement in prostate cancer patients, and the images contain more valuable textural features. ADC values objectively reflect the degree of diffusion of water molecules in biological tissue and correlate with the malignancy of the tumor, avoiding the penetration effect of DWI due to the very long T2 decay time of the tissue. The combination of T2WI and ADC allows for more accurate and comprehensive tumor information to be obtained. In our study, the combined sequence of T2WI and ADC showed better performance in predicting 3-year PFS in PCa patients than the model with the sequence alone, with the highest AUC in both the training and validation groups.

Age, pretreatment PSA levels, TNM stage and Gleason score all have an impact on the prognosis of PCa. In this study, using univariate Cox risk regression analysis, the clinical T stage, pretreatment PSA level and Gleason score were found to have a statistically significant impact on the prognosis of PCa; age was not. Some scholars (31) conducted an epidemiological survey and analysis on the effect of age on survival, comparing the effect of different age segments on survival. The results showed that patients in the younger group survived longer, and the difference was statistically significant, but it has also been shown (32) that age is not an influential factor in the prognosis of prostate cancer. Our findings do not support age as an independent influential factor in the prognosis of patient survival. We also did a simple Kendall correlation analysis of the effect of T-stage, Gleason score and number of lesions on the patient’s PSA levels and found that the three clinical factors were positively correlated with PSA levels and that the Gleason score correlated more significantly with them. This suggests that the PSA level is also increased with an increase in Gleason score. The PIRADS v2 score is currently the most widely used and internationally recognized MRI reporting system for the prostate. de Cobelli O et al. (33) found a significant association between PIRADS score and GS escalation, ECE, unfavorable prognosis and large tumor volume: increasing with increasing PIRADS score. We will also include PI-RADS in a follow-up study to discuss its relevance to the prognosis of prostate cancer.

The concept of adequate mutual agreement between genitourinary radiologists has been a key point of discussion. mpMRI has changed the paradigm of prostate cancer detection, characterization and management, refining treatment planning and patient selection for active surveillance, and assessing post-treatment outcomes, but the interpretation of mpMRI remains difficult and has substantial inter-reader variability, leading to the development of the original (v.1) and updated (v.2 and 2.1) versions of the PI-RADS development. Del Giudice et al. (34)demonstrated that Vesical Imaging-Reporting and Data System (VI-RADS) provides a standard method for radiologists in the acquisition, interpretation and reporting of MRI of bladder cancer. Despite the existence of two very independent diagnostic goals between PI-RADS and VI-RADS, these standard certainties share the common goal of pursuing a higher reliability of diagnostic findings in the reader than a purely subjective interpretation of MRI sequences, which also provides ample evidence of the importance of rigorous monitoring for a high degree of inter-reader agreement between different AI and radiomic features.

Many studies have attempted to combine imaging histology with clinical parameters to improve the predictive power of the model. The nomogram was developed by Yu et al. (35) With the combination of radiomics features and clinical parameters was able to predict peritoneal metastases in ovarian cancer preoperatively well, and its efficacy was superior to that of a single model with radiomics and the clinic. We also developed a hybrid model to plot a nomogram combining Rad scores and important clinical features for the assessment of 3-year PFS in PCa patients. The hybrid model showed superior predictive performance for 3-year PFS prediction compared to the radiomics model alone. The ROC curve analysis also validates this result. Our study also found that the Rad score could be used as a marker to distinguish between low- and high-risk patients. Patients with higher Rad scores are at greater risk of progression and have shorter PFS. These results provide new insights into future treatment options for patients with PCa. For example, patients at high risk of progression may consider a combination of early multiple treatments; conversely, patients at low risk of progression may opt directly for surgery, local radiotherapy or even monitoring, thus avoiding ineffective or excessive treatment and disease progression due to delays in effective treatment. Therefore, the Rad score can be used as a valid biomarker to improve the prognosis of patients with PCa.

There are some limitations to our study. First, this is a single-center, retrospective study with some possible bias in the selection of patients, which will be validated in future research through multicenter, prospective studies to provide more reliable evidence for clinical application. Second, the follow-up period was relatively short, and longer follow-up is needed to predict 5-year and 10-year progression-free survival, which can be used as part of our follow-up study. Third, radiomics seeks to find the most valuable features in a variety of data, and we only analyzed T2WI and ADC images without adding dynamic enhancement images to the analysis. Multiparametric data analysis may help improve the quality of the model. Fourth, some important protein and gene biomarkers associated with PCa progression were not considered for the features we extracted from the MRI. Finally, our ROIs were obtained by manual segmentation by radiologists, with subjective observer bias, and a reliable and robust automated segmentation method should be further developed to address this issue.

## Conclusion

In summary, in this study, we retrospectively analyzed the relationship between MRI radiomics features and progression-free survival in patients with prostate cancer confirmed by biopsy puncture or surgical pathology and analyzed the feasibility of imaging histology for the assessment of progression-free survival. The radiomics features extracted by MRI provide a highly accurate, noninvasive, easy-to-perform, real-time method for preoperatively predicting progression-free survival in prostate cancer patients. Multiple sequence combination models are superior to single sequence models. We developed a nomogram to provide a noninvasive, individualized tool for the stratified management of prostate cancer patients to support clinical decision-making. Although there are some limitations to our study, we have provided a means of assessing the preoperative prediction of tumor progression in prostate cancer patients, compensating for the shortcomings of conventional imaging.

## Data availability statement

The raw data supporting the conclusions of this article will be made available by the authors, without undue reservation.

## Ethics statement

The studies involving human participants were reviewed and approved by Ethics Committee of the Affiliated Hospital of Inner Mongolia Medical University. The patients/participants provided their written informed consent to participate in this study.

## Author contributions

YJ and SQ substantial contributions to the conception or design of the work; or the acquisition, analysis or interpretation of data for the work. HW and AL drafting the work or revising it critically for important intellectual content. TZ, HH, JR and ZY provide approval for publication of the content. YG and FH agree to be accountable for all aspects of the work in ensuring that questions related to the accuracy or integrity of any part of the work are appropriately investigated and resolved. All authors contributed to the article and approved the submitted version.

## Funding

This article was funded by the Inner Mongolia Autonomous Region Fund of Natural Science (2021MS08026); the General Program of Inner Mongolia Medical University (YKD2021MS045); Inner Mongolia Medical University College Students Science and Technology Innovation “Talent Cultivation”(YCPY2021088); Inner Mongolia Autonomous Region Fund of Natural Science(2022SHZR2186).

## Conflict of interest

Author SQ and JR were employed by GE Healthcare.

The remaining authors declare that the research was conducted in the absence of any commercial or financial relationships that could be construed as a potential conflict of interest.

## Publisher’s note

All claims expressed in this article are solely those of the authors and do not necessarily represent those of their affiliated organizations, or those of the publisher, the editors and the reviewers. Any product that may be evaluated in this article, or claim that may be made by its manufacturer, is not guaranteed or endorsed by the publisher.
