# Corrigendum: MRI radiomics predicts progression-free survival in prostate cancer

**DOI:** 10.3389/fonc.2022.1125641

**Published:** 2023-01-13

**Authors:** Yushan Jia, Shuai Quan, Jialiang Ren, Hui Wu, Aishi Liu, Yang Gao, Fene Hao, Zhenxing Yang, Tong Zhang, He Hu

**Affiliations:** ^1^ Affiliated Hospital, Inner Mongolia Medical University, Hohhot, China; ^2^ Department of Pharmaceuticals Diagnosis, GE Healthcare (China), Shanghai, China; ^3^ Department of Radiology, Affiliated Hospital of Inner Mongolia Medical University, Hohhot, China

**Keywords:** prostate cancer, radiomics, progression-free survival, magnetic resonance imaging, predictions

In the published article, there was an error in affiliation 3. Instead of “Department of Radiology, Inner Mongolia International Hospital, Hohhot, China”, it should be “Department of Radiology, Affiliated Hospital of Inner Mongolia Medical University, Hohhot, China”.

The authors apologize for this error and state that this does not change the scientific conclusions of the article in any way. The original article has been updated.

